# Segmented Helical Structure of the Neck Region of the Glycan-Binding Receptor DC-SIGNR

**DOI:** 10.1016/j.jmb.2009.10.006

**Published:** 2009-12-11

**Authors:** Hadar Feinberg, Cynthia K.W. Tso, Maureen E. Taylor, Kurt Drickamer, William I. Weis

**Affiliations:** 1 Department of Structural Biology, Stanford University School of Medicine, Stanford, CA 94305, USA; 2 Division of Molecular Biosciences, Department of Life Sciences, Imperial College London, London SW7 2AZ, UK

**Keywords:** CRD, carbohydrate-recognition domain, DC-SIGNR, crystal structure, glycan-binding receptor, oligomerization, polymorphisms

## Abstract

Carbohydrate-recognition domains (CRDs) in the glycan-binding receptors DC-SIGN (dendritic-cell-specific intercellular adhesion molecule 1-grabbing nonintegrin; CD209) and DC-SIGNR (DC-SIGN-related receptor, also known as L-SIGN and variously designated CD209L and CD299) are projected from the membrane surface by extended neck domains containing multiple repeats of a largely conserved 23-amino-acid sequence motif. Crystals of a fragment of the neck domain of DC-SIGNR containing multiple repeats in which each molecule extends through multiple unit cells, such that the observed crystallographic asymmetric unit represents one repeat averaged over six repeats of the protein, have been obtained. The repeats are largely α-helical. Based on the structure and arrangement of the repeats in the crystal, the neck region can be described as a series of four-helix bundles connected by short, non-helical linkers. Combining the structure of the isolated neck domain with a previously determined overlapping structure of the distal end of the neck region with the CRDs attached provides a model of the almost-complete extracellular portion of the receptor. The results are consistent with previous characterization of the extended structure for the isolated neck region and the extracellular domain. The organization of the neck suggests how CRDs may be disposed differently in DC-SIGN compared with DC-SIGNR and in variant forms of DC-SIGNR assembled from polypeptides with different numbers of repeats in the neck domain.

DC-SIGN (dendritic-cell-specific intercellular adhesion molecule 1-grabbing nonintegrin; CD209) and DC-SIGNR (DC-SIGN-related receptor, also known as L-SIGN and variously designated CD209L and CD299) are glycan-binding receptors of the immune system.^1^ DC-SIGN is expressed on dendritic cells and some types of macrophages,^2,3^ while DC-SIGNR is expressed on sinusoidal endothelial cells and in the placenta.^4^ Similar to mannose-binding protein, the macrophage mannose receptor, and langerin, these receptors contain C-type carbohydrate-recognition domains (CRDs).^5^ Although each of these receptors binds mannose and related monosaccharides, they have differing specificities for oligosaccharides and for surfaces of microorganisms.^1,6^ Both DC-SIGN and DC-SIGNR bind high mannose oligosaccharides found on viral envelope glycoproteins and can participate in viral infection either directly by facilitating viral entry into a target cell or indirectly by presenting viruses to target cells.^7–10^ DC-SIGN also binds fucose-containing glycans, which can serve in recognition of parasites and in binding to T cells.^11,12^

The CRDs in DC-SIGN and DC-SIGNR tetramers are projected at the ends of extended neck regions formed from a series of conserved but not identical sequences of 23 amino acids.^13^ The neck portions of DC-SIGN and DC-SIGNR function as independent tetramerization domains.^14^ Circular dichroism analysis indicates that the necks contain extensive α-helical regions, and the sequences of the repeats show evidence of a heptad repeat, indicating a possible coiled-coil structure. However, each repeat also contains a conserved proline residue that would interrupt a helical structure, so the overall structure of the neck repeats has been unclear. Several lines of evidence suggest that the CRDs in DC-SIGN and DC-SIGNR are not held in a fixed position and that they can reorient to engage with ligands,^15,16^ but a more detailed understanding of the neck region is needed to explain its role in positioning the CRDs.

Common polymorphisms in the DC-SIGNR gene result in polypeptides with different numbers of neck repeats.^17,18^ Various genotypes at the DC-SIGNR locus result in DC-SIGNR homo- and hetero-oligomers with different polypeptide compositions in different individuals.^19^ Genetic studies indicate that these differences may correlate with susceptibility to infections.^17,18^ A better understanding of how length differences are accommodated in the structure of the DC-SIGNR neck would provide insight into their effects on disposition of ligand-binding sites and thus the ability of different forms of the receptor to interact with viruses and other pathogens.

### Characterization and crystallographic analysis of a truncated neck domain

In order to examine the structure of the neck portion of DC-SIGNR, we investigated several truncated forms of the neck region as possible crystallization targets. In previous studies, the extracellular domain was truncated only at the C-terminus by replacing the CRD with a simple two-histidine tag, which allowed efficient binding of the tetrameric neck to chelated nickel columns through the resulting cluster of eight histidine residues.^14^ Further truncated forms were generated by removing the N-terminal non-repeat region or by removing this region and the first half of the first repeat unit (Fig. 1a). The latter truncation site was based on the presence of a protease-sensitive site immediately after Leu104 in the full-length neck, suggesting that the first part of the neck may be conformationally flexible when released from the membrane and in the absence of the normal N-glycosylation in this repeat.^19^ Gel filtration suggested that the purified truncated fragment is tetrameric, and differential scanning calorimetry demonstrated that it retains the stability of the full-length DC-SIGNR neck region, with a melting temperature of 81.7 °C compared with the measured value of 80.1 °C for the full-length neck (Fig. 1b and c). Thus, the N-terminal region that has been removed does not contribute significantly to the stability of the tetramer.

Crystals of the truncated neck fragment were obtained following screening of a sparse matrix of crystallization conditions. After refining the crystallization conditions, a diffraction data set was measured (Table 1). The crystals are in space group *P*42_1_2, with unit cell dimensions *a* = 34.2 Å, *b* = 34.2 Å, and *c* = 36.7 Å. The asymmetric unit of these crystals cannot contain the full protein used for crystallization. With only one repeat in the asymmetric unit, the calculated Matthews coefficient is 2.0, with a solvent content of 40%. Lower-symmetry space groups were considered, but both data statistics and the final solution indicated that the symmetry is in fact *P*42_1_2. Several crystals were collected, washed with a solution similar to the mother liquor but with a higher concentration of polyethylene glycol, and analyzed by SDS-PAGE to confirm that the polypeptide was completely intact and not degraded during crystallization.

The structure was determined by multiwavelength anomalous dispersion phasing using crystals soaked in Pb(CH_3_COO)_2_ (Table 1). An anomalous-difference Patterson map showed strong peaks corresponding to a single heavy atom site. The site was found using the program Phenix^22^ and was refined in CNS^23^ to a figure of merit of 0.8. The phases were improved with density modification to give a map at 2.5-Å resolution that showed clear α-helices. A polyalanine model was built into the map using Coot,^24^ and rigid-body refinement against the remote data set was performed with CNS.

Within the unit cell, four molecules interact around the crystallographic 4-fold axis to generate a four-helix bundle (Fig. 2a). This bundle packs against a 2-fold-symmetry-related antiparallel four-helix bundle that also runs along the *c* axis (Fig. 2b). The four-helix bundles are translated by various numbers of repeats along the *c* axis throughout the crystal. This arrangement produces a unit cell with an asymmetric unit one repeat long that is an average of six nearly identical repeats.

Simulated annealing of the partial polyalanine model with Phenix clarified the density for some missing residues. Side chains that are conserved among the six repeats were gradually added during several rounds of positional and isotropic temperature factor refinement alternating with manual model adjustment, increasing the resolution to 2.3 Å. This partial model was rigid-body refined against the native data set. Identical reflections were chosen as test set to calculate *R*_free_ to be consistent with the heavy atom data. The model was further refined in Phenix to 2.2 Å. Completely conserved residues were given occupancy of 1.0, and side chains at the non-conserved positions were built with occupancy assigned based on their frequency in repeats 2 through 7 (Fig. 1a): proline with occupancy of 0.83 and serine with occupancy of 0.17 at position 1 as well as glutamate with occupancy of 0.17, glutamine with occupancy of 0.33, and arginine with occupancy of 0.5 at position 15, resulting in the final refined model described in Table 2.

The final model contains all 23 residues of the repeat and 21 water molecules. The electron density map did not show any features corresponding to repeat 8, which is the least conserved of the repeats. In addition, the 4-fold symmetry places the α-helices in close proximity, such that the large side chain of a phenylalanine residue at position 20 of repeat 8 (Phe261 in Fig. 1a) would not be accommodated. Both the lack of electron density for the nonconserved side chains in repeat 8 and the positions of the α-helices suggest that repeat 8 is disordered in the crystal or that diffraction from the nonconserved structure is obscured by the signal from the other six, almost identical, repeats. Thus, there may be gaps of one or more unit cells between successive tetramers along the *c* axis to accommodate a disordered repeat 8 and the extra residues at the N- and C-termini of the truncated protein.

### Structure and linkage of neck repeats

The structure of the neck unit representing repeats 2 through 7 of the neck of DC-SIGNR is predominantly that of a four-helix bundle. The final model in the asymmetric unit is one repeat long (Fig. 2a) and, applying the 4-fold symmetry present in space group *P*42_1_2, produces the four-α-helical bundle (Fig. 2c). The sequence of amino acids in the helical portions of the repeats follows the expected heptad pattern, with hydrophobic amino acids at the *a* and *d* positions (Fig. 1a). These hydrophobic residues are positioned toward the center of the bundle and pack against one another (Fig. 2c). There are also multiple hydrophobic, hydrogen-bond, and ionic interactions between side chains within each subunit and between subunits (Fig. 2d). The side chain of Lys17 is not well defined in the map, but it is possible that another rotamer of this residue would allow it to interact with Glu12 (Fig. 2d) to make the interaction proposed by Tabarani *et al*.^25^

Translation of the α-helical bundle produces a polymer of repeats in which residues Pro1 and Leu23 at the N- and C-termini are covalently linked to residues Leu23 and Pro1 from symmetry-related molecules (Fig. 2e). An α-helix of one repeat is connected to the α-helix of another repeat that is translated along the 4-fold axis and rotated 90° about the same axis. The side chain of Pro1 is positioned toward the center of the bundle, packing against the main chain of Pro1, Glu2 and Lys3, and the side chain of Leu6 from a symmetry-related monomer (Fig. 2e).

A model for the structure of the extended neck region was initially built by applying rotational symmetry operations and translations to the one repeat in the asymmetric unit, extending the four-helical bundle to represent repeats 2–8 (Fig. 3a). The neck is seen to adopt an elongated form, composed of α-helical repeats that form four-helical bundles that are separated by short kinks around the proline residues at position 1 of each repeat.

Previous hydrodynamic studies were conducted on a fragment of DC-SIGN that included all of the repeats plus the less conserved 15-residue extension at the N-terminal end that links the repeat region to the membrane.^14^ Using the crystallographic data, we constructed an eight-repeat structure as a model for this fragment, lacking just 8 N-terminal residues and the C-terminal Gly–His–His tag. Calculation of the hydrodynamic properties of the modeled protein using HydroPro 7c^26^ gave values for the sedimentation coefficient of *s*_20,w_ = 3.41 S and the diffusion coefficient of *D*_20,w_ = 3.88 × 10^− ^^7^ cm^2^/s, in very close agreement with the measured values of *s*_20,w_ = 3.37 S and *D*_20,w_ = 3.80 × 10^− ^^7^ cm^2^/s. Thus, the model for the neck derived from the repeat structure in the crystal is consistent with the behavior of the isolated neck as a rigid, extended structure.

### Structure of the extracellular domain of DC-SIGNR

A previously published structure of a fragment of DC-SIGNR [Protein Data Bank (PDB) ID 1XAR] comprises the C-terminal part of repeat 8 of the neck with the CRD in a tetrameric form (Fig. 3b). Comparison between the four-α-helical bundle of repeat 8 in this structure and the four-helical bundle seen here in the neck repeats reveals that the helices are positioned similarly up to residue Leu254 (extracellular domain numbering). Residues in positions 253 and 254 are present in both the tetramer-CRD and neck domain structures, and C^α^ superposition of the four copies of these two residues in the two structures gives an rmsd of 0.67 Å. In contrast, the positions of residues in the C-terminal portion of repeat 8, comprising residues 255–264, are slightly different in the tetramer-CRD structure and the neck repeats. The α-helices are splayed apart from one another, which breaks the 4-fold symmetry to form a dimer of dimers. This difference accommodates residue Phe261, corresponding to position 20 in repeat 8 (Figs. 1a and 3b) and places the CRDs in the orientations seen in the tetramer structure. C^α^ superposition of four copies of these eight residues in the two structures gives an rmsd of 1.53 Å.

A model of the almost-complete extracellular domain was generated by superimposing the C^α^ positions of the four copies of residue Leu254 from the tetramer-CRD onto those of the four-helix bundle of the neck. The final model comprises repeats 2–7 and the N-terminal portion of repeat 8 up to residue 253 taken from the neck structure as well as the C-terminal part of repeat 8 starting at residue 254 and the CRD as seen in the repeat 8–CRD tetramer structure (Fig. 3c). The side chains in the N-terminal portion of repeat 8 were changed to correspond to the natural sequence, with Asp243, Gln244, Gln247, and Gln249, at positions 2, 3, 6, and 8 of this repeat (Fig. 1a).

The structural model in Fig. 3c is consistent with other physical characterizations of the extracellular portion of DC-SIGNR. The model represents almost exactly the portion of the extracellular domain of DC-SIGNR that was previously characterized in hydrodynamic experiments, lacking only three N-terminal and three C-terminal residues.^19^ The program HydroPro 7c^26^ was used to predict values for the sedimentation coefficient of *s*_20,w_ = 5.22 S and the diffusion coefficient of *D*_20,w_ = 3.37 × 10^− 7^ cm^2^/s, which compare well with the measured values of *s*_20,w_ = 5.36 S and *D*_20,w_ = 3.45 × 10^− 7^ cm^2^/s. The fact that the measured values are slightly higher than the predicted values may reflect the fact that the hydrodynamic modeling program is based on a rigid molecule, while in fact the CRDs are not fully fixed in position. The presence of breaks in the α-helical structure, resulting in relatively short helices of 18 residues abutting non-helical regions at each end, means that there is a much larger end effect in measurements of circular dichroism than would be expected for a continuously helical neck.^27^ Thus, the segmented nature of the helices accounts for the low estimate of 40% helical content obtained using global fitting algorithms.^14^

Force–distance measurements have previously been used to study a version of the extracellular domain of DC-SIGN corresponding to the fragment of DC-SIGNR modeled in Fig. 3c with an N-terminal His_6_ tag for immobilization on a membrane surface.^16^ The results indicate that the protein extends 328 Å in the absence of interaction with ligand and that it can resist compression by an opposing membrane surface. The rigidity of the structure is consistent with the structure of the neck in DC-SIGNR, in which the four polypeptides interact extensively throughout their length, making the tetramer resistant to bending. However, when the CRDs engage with ligands on an apposed surface, there is a conformational change, probably due to reorientation of the CRDs, which results in a reduction of the overall length to approximately 280 Å. Parallel studies indicate that the extracellular domain of DC-SIGNR has similar dimensions and undergoes similar conformational changes (S. Menon, M. E. Taylor, K. Drickamer, and D. A. Leckband, unpublished results). The overall length of the model in Fig. 3c is 265–270 Å, which suggests that the disposition of the CRDs in the model resembles that of the structure engaged with ligand. A model for the DC-SIGNR tetramer with bound oligosaccharide ligand was obtained by superimposing the crystal structure of the CRD complexed with Man_3_GlcNAc_2_^6^ (PDB ID 1K9J) onto the CRDs in the model in Fig. 3c and then superimposing the trimannose core of Man_9_GlcNAc_2_ onto the equivalent part of Man_3_GlcNAc_2_ (Fig. 3d). The resulting model shows that the reducing ends of the oligosaccharides would be oriented upward, consistent with the proposed arrangement when interacting with glycans on a membrane surface.

Modeling based on recent small-angle X-ray scattering measurements of the DC-SIGN extracellular domain is also roughly consistent with the overall length of the molecule, but the molecular envelope derived from the scattering data suggested an alternative rigid positioning of the CRDs in a more extended structure.^25^ Such an extended structure could be stabilized by anomalous inter-CRD disulfide bonds that are present when the protein from the bacterial expression system is prepared following the published procedure^13^ unless subjected to additional steps of purification (C. Tso, M. E. Taylor, and K. Drickamer, unpublished observations). Nevertheless, the surface force measurements of DC-SIGN and DC-SIGNR are consistent with the idea that there exists a more extended conformation of the extracellular domain that can change to the conformation seen in the crystals. In the light of these considerations, it would be interesting to assess how well the crystallographically observed neck structure would fit the scattering envelope.

### Variations in DC-SIGN and DC-SIGNR structures

Although DC-SIGN and DC-SIGNR are very similar in amino acid sequence and polypeptide organization, they do display important differences in the neck regions as well as in the CRDs. Some of the differences in the neck region are in the heptad residues of the helical regions, particularly at position 6 in the repeat. The presence of glutamine at position 6 in repeats 6 and 7 in the neck of DC-SIGN, rather than leucine in DC-SIGNR, correlates with the higher stability of the DC-SIGNR neck in thermal denaturation studies. Chimeric necks have the stability properties of DC-SIGNR when they contain leucine at these positions but behave similarly to DC-SIGN when glutamine is present.^14^ A further key difference is in the final neck repeat, in which the phenylalanine residue at position 20 of the repeat, corresponding to residue 261 in the overall sequence of DC-SIGNR, is replaced by a valine residue in DC-SIGN. The smaller size of the valine side chain means that the helices of the final repeat could be packed similarly to those in the remainder of the neck and the 4-fold symmetry could extend all the way to the end of the molecule. This difference could shift the orientation of the CRDs slightly upward and change the relative disposition of the glycan-binding sites, and it might also explain the difference between the model of DC-SIGNR derived here and that of DC-SIGN in the work of Tabarani *et al*.^25^

The structure of the neck of DC-SIGN provides some important insights into how neck length polymorphisms in the DC-SIGNR gene affect the structure of the protein and how these might in turn affect interaction with ligands, such as viral membrane glycoproteins. First, the slight splaying apart of the C-terminal portion of the helical region in the final neck repeat places the CRDs somewhat to the side of rather than at the ends of the helices. As a result, in hetero-oligomers containing a mixture of polypeptides with different numbers of neck repeats, CRDs on the shorter polypeptides would project out from the side of the neck and would thus be accommodated adjacent to a further neck repeat in the longer polypeptide (Fig. 3e). Second, one of the most striking features of the model in Fig. 3e is the twisting of the neck, resulting from the pitch of the supercoiling in the segments of the four-helical bundle and the way that the proline linkers join these segments. As a consequence of this arrangement, the position of the C-terminal end of each repeat is rotated 90° from the previous repeat along the polypeptide moving away from the membrane surface. Therefore, in a hetero-oligomer, the position of a CRD on a shorter polypeptide would be rotated 90° around the neck as well as translated one repeat length closer to the membrane and would thus be below the CRD in an adjacent, longer repeat rather than next to it. The resulting difference in the orientation of the glycan-binding sites could dramatically affect the relative affinities of homo-oligomers and hetero-oligomers for surface glycoproteins on different enveloped viruses.

### PDB accession number

Coordinates and structure factors have been deposited in the PDB with access number 3JQH.

## Figures and Tables

**Fig. 1:**
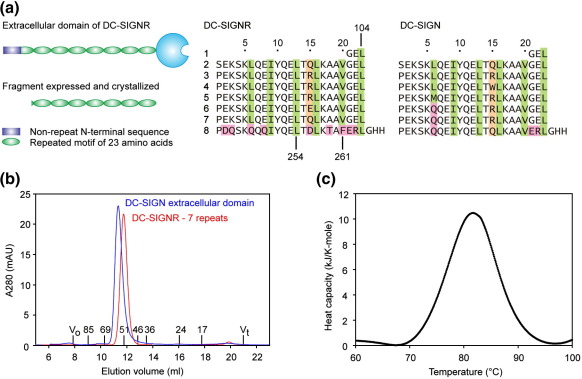
(a) Summary of the organization of DC-SIGNR and the fragment expressed and crystallized along with the sequence of this fragment and the corresponding portion of DC-SIGN. Hydrophobic residues packing with one another in the four-α-helical bundles are marked in green. Position 15, at which residues differ in different repeats, is highlighted in beige. Residues that differ in the final repeat and glutamine residues in heptad repeat positions are shaded in pink. Lines indicate residue numbers based on the full-length sequence of DC-SIGNR (SwissProt accession number Q9H2X3). (b) Gel-filtration profile for the expressed fragment of DC-SIGNR superimposed on the profile for the previously described full extracellular domain of DC-SIGN.^14^ (c) Differential scanning calorimetry of the expressed fragment showing a denaturation temperature of approximately 81.7 °C, which is close to the value of 80.1 °C obtained under identical conditions for the full extracellular domain.^14^

**Fig. 2:**
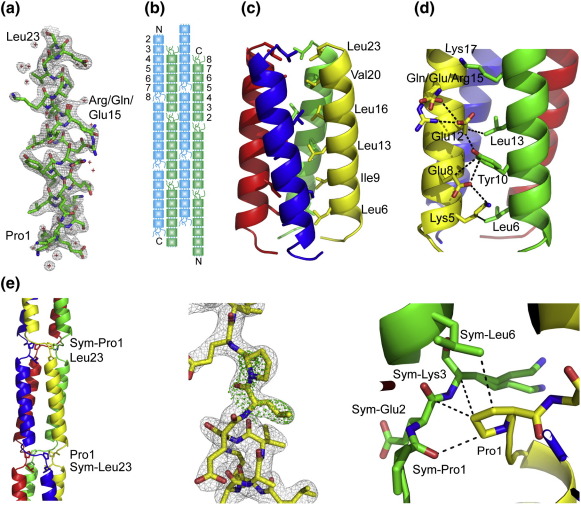
Structure of the 23-amino-acid repeat motif. (a) The 23-amino-acid model in the asymmetric unit shown with the final 2*F*_o_ − *F*_c_ electron density map (1.0σ contour). (b) Proposed packing of eight neck repeats in the crystals, with molecules running in opposite directions illustrated in blue and green. (c) Four-α-helical bundle formed by the 4-fold symmetry of the space group. The protein is shown in cartoon representation, with side chains of hydrophobic residues positioned toward the center of the bundle shown in stick representation. (d) Representative interactions between side chains in the four-helix bundle. (e) Connections between 23-amino-acid repeat motifs through non-helical segments. Left, one repeat is shown connected at the N- and C-termini to symmetry-related repeats. Center, close-up of the Leu23–Pro1 connection between successive repeats. The 2*F*_o_ − *F*_c_ electron density map (1.0σ contour) is shown as a gray mesh, and an *F*_o_ − *F*_c_ map made by omitting residues Pro1 and Leu23 from the model is shown in green (3.0σ contour). Right, interactions of Pro1 with symmetry-related monomers. All molecular graphic figures were prepared with PyMol (http://www.pymol.org).

**Fig. 3:**
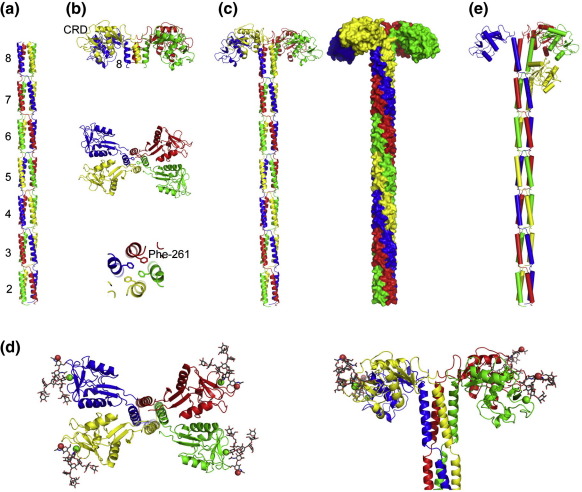
Construction of a model of the full expressed fragment of the extracellular domain. (a) Seven four-helical bundles made by applying rotational and translational symmetry operations to the model in the asymmetric unit of the neck domain. (b) Top and side views of the previously published structure of the tetramer of a fragment containing the terminal repeat and CRDs (PDB ID 1XAR). Monomer A is shown in green, monomer B is shown in red, monomer C, which is a 2-fold symmetry mate of A, is shown in blue, and monomer D, a 2-fold symmetry mate of B, is shown in yellow. The side chain of residue Phe261 is shown in stick representation. (c) Model of much of the extracellular domain of DC-SIGNR, including neck repeats 2 to 8 and the CRD, created by superposition of the structures in (a) and (b). (d) Model of the tetrameric DC-SIGNR extracellular domain complexed with a Man_9_GlcNAc_2_ oligosaccharide. Green spheres represent Ca^2+^. The red sphere corresponds to the reducing end of the oligosaccharide, which would be attached to a lipid membrane in the force–distance measurements. Flexibility in the positions of these sites of attachment, resulting from different orientations of the CRDs, would be required for binding to oligosaccharides on viral glycoproteins in multiple orientations. (e) Model of a hetero-oligomer containing a subunit one neck repeat shorter than the others showing the relative positions of the CRDs.

**Table 1:** Crystallographic data statistics

Data	Native	PbAc_2_ peak	PbAc_2_ remote	PbAc_2_ inflection
Wavelength	0.97945	0.94894	0.87309	0.94992
Space group	*P*42_1_2	*P*42_1_2	*P*42_1_2	*P*42_1_2
Unit cell lengths (Å)				
* a*	34.17	32.60	32.60	32.64
*b*	34.17	32.60	32.60	32.64
*c*	36.72	36.11	36.15	36.21
Resolution (last shell) (Å)	36.7–2.2 (2.32–2.20)	36.1–2.3 (2.38–2.30)	36.1–2.3 (2.38–2.30)	36.2–2.3 (2.38–2.30)
*R*_sym_ (last shell)^a^	6.1 (12.6)	8.3 (25.9)	7.6 (25.8)	7.9 (39.8)
Mean [(*I*)/sd(*I*)]	32.0 (26.3)	27.8 (14.5)	29.2 (14.2)	33.6 (10.0)
% completeness (last shell)	99.9 (100)	99.0 (99.0)	98.9 (99.0)	98.8 (97.9)
Average multiplicity	14.7 (15.4)	24.2 (25.2)	24.0 (25.3)	24.1 (24.5)

Crystals of the human DC-SIGNR neck repeats were grown at 18 °C using the hanging-drop method (0.6 μl of protein to 0.6–1.2 μl of reservoir buffer in a drop). The protein solution contained 2.8 mg/ml of protein, 10 mM Tris–Cl, pH 8.0, and 25 mM NaCl. The reservoir solution contained 9% polyethylene glycol 6000, 1.25 M NaCl, and 0.1 Bis-Tris, pH 6.5. Native crystals were transferred to a solution containing 30% polyethylene glycol 6000, 0.05 M Bis-Tris, pH 6.5, and 1 M NaCl and frozen in liquid nitrogen for data collection. For heavy atom soaks, crystals were washed in a solution containing 30% polyethylene glycol 6000, 0.05 Mes buffer, pH 6.5, and 1 M NaCl by moving the crystals twice from one drop to another, resulting in exchange of the Tris buffer with Mes and increased concentration of the polyethylene glycol. Washed crystals were transferred to the same solution with the addition of 1 mM PbAc_2_ and were frozen the next day in liquid nitrogen for data collection. Diffraction data were measured at 100 K on a MAR 325 CCD detector at the Stanford Synchrotron Radiation Laboratory beamline 11-1. The native data set was processed with MOSFLM and SCALA,^20^ and the heavy atom derivative was processed with HKL2000.^21^ A multiwavelength anomalous diffraction data set was collected for the PbAc_2_ crystal (peak, high remote, and inflection).

a *R*_sym_ = 100 × ∑_*h*_∑*_i_*(|*I*_*i*_(*h*)| − |〈*I*(*h*)〉|)/∑*_h_*∑*_i_I_i_*(*h*), where *I*_*i*_(*h*) is the observed intensity and 〈*I*(*h*)〉 is the mean intensity obtained from multiple measurements.

**Table 2:** Crystallographic refinement statistics

Residues included in the final model	23
*R*_free_^a^	21.6
*R*_work_^a^	19.4
Average temperature factor (Å^2^)	65.8
Bond length rmsd (Å)	0.007
Angle rmsd (°)	0.96
Ramachandran plot (% in each region)^b^	
Preferred	100
Allowed	0
Outliers	0

a *R* and *R*_free_ = 100 × ∑||*F*_o_| − |*F*_c_||/∑|*F*_o_|, where |*F*_o_| is the observed structure factor amplitude and |*F*_c_| is the calculated structure factor amplitude for the working and test sets, respectively.

b As defined in Coot.
